# Novel adiponectin-resistin (AR) and insulin resistance (IR_AR_) indexes are useful integrated diagnostic biomarkers for insulin resistance, type 2 diabetes and metabolic syndrome: a case control study

**DOI:** 10.1186/1475-2840-10-8

**Published:** 2011-01-21

**Authors:** Cia-Hin Lau, Sekaran Muniandy

**Affiliations:** 1 Department of Molecular Medicine, Faculty of Medicine, University of Malaya, 50603 Kuala Lumpur, Malaysia

## Abstract

**Background:**

Adiponectin and resistin are adipokines which modulate insulin action, energy, glucose and lipid homeostasis. Meta-analyses showed that hypoadiponectinemia and hyperresistinemia are strongly associated with increased risk of insulin resistance, type 2 diabetes (T2DM), metabolic syndrome (MS) and cardiovascular disease. The aim of this study was to propose a novel adiponectin-resistin (AR) index by taking into account both adiponectin and resistin levels to povide a better indicator of the metabolic homeostasis and metabolic disorders. In addition, a novel insulin resistance (IR_AR_) index was proposed by integration of the AR index into an existing insulin resistance index to provide an improved diagnostic biomarker of insulin sensitivity.

**Methods:**

In this case control study, anthropometric clinical and metabolic parameters including fasting serum total adiponectin and resistin levels were determined in 809 Malaysian men (208 controls, 174 MS without T2DM, 171 T2DM without MS, 256 T2DM with MS) whose ages ranged between 40-70 years old. Significant differences in continuous variables among subject groups were confirmed by ANCOVA or MANCOVA test using 1,000 stratified bootstrap samples with bias corrected and accelerated (BCa) 95% CI. Spearman's rho rank correlation test was used to test the correlation between two variables.

**Results:**

The AR index was formulated as 1+log_10_(R_0_)-log_10_(A_0_). The AR index was more strongly associated with increased risk of T2DM and MS than hypoadiponectinemia and hyperresistinemia alone. The AR index was more strongly correlated with the insulin resistance indexes and key metabolic endpoints of T2DM and MS than adiponectin and resistin levels alone. The AR index was also correlated with a higher number of MS components than adiponectin and resistin levels alone. The IR_AR _index was formulated as log_10_(I_0_G_0_)+log_10_(I_0_G_0_)log_10_(R_0_/A_0_). The normal reference range of the IR_AR _index for insulin sensitive individuals was between 3.265 and 3.538. The minimum cut-off values of the IR_AR _index for insulin resistance assessment were between 3.538 and 3.955.

**Conclusions:**

The novel AR and IR_AR _indexes are cost-effective, precise, reproducible and reliable integrated diagnostic biomarkers of insulin sensitivity for screening subjects with increased risk of future development of T2DM and MS.

## Background

The world prevalence of diabetes among adults will be 6.4%, affecting 285 million adults, in year 2010, and will increase to 7.7%, and 439 million adults by year 2030 [1]. Malaysia is listed as the top 10 countries with the highest prevalence of diabetes in recent global estimate of the prevalence of diabetes for years 2010 and 2030 [1]. In addition, a recent nationwide survey showed that Malaysia has a much higher prevalence of metabolic syndrome (MS) compared with other Asian countries [2]. Insulin resistance is a prerequisite root factor for development of type 2 diabetes (T2DM) [3]. It is also the most unifying parameter to characterize the pathophysiology of the MS [3]. The MS drives the twin global epidemics of T2DM and cardiovascular disease [4]. T2DM itself is accompanied by increased risk for cardiovascular disease which is aggravated by the concomitant risk factors of the MS [4]. Adiponectin [5] and resistin [6] hormones are thought to link T2DM and MS with cardiovascular risk.

Adiponectin is an adipocyte-secreted polypeptide hormone with molecular weight 30 kDa (244 amino acids) which modulates a number of metabolic processes, and regulates insulin sensitivity and energy homeostasis, as well as glucose and lipid metabolism [7]. The hormone plays a principal role in the suppression of the metabolic derangements that may result in insulin resistance, T2DM, MS, and cardiovascular disease [5,8,9].

Resistin is a macrophage-derived signalling polypeptide hormone with molecular weight 12.5 kDa and its length is 108 amino acids in humans [10]. In contrast with adiponectin, resistin has low circulating levels [10]. However, the blood circulating levels of resistin have been shown to be up-regulated in subjects with insulin resistance, T2DM, MS, and cardiovascular disease [6,11].

The concurrence of hypoadiponectinemia [5,8,9] and hyperresistinemia [6,10] in subjects with insulin resistance, T2DM and MS risk are well-established. A significant inverse correlation between adiponectin and resistin levels has also been reported in the literatures [12,13]. The overall structure of multimeric assembly or oligomerization of resistin is similar to that of adiponectin [14]. Taking these studies together, it may be speculated that adiponectin and resistin share a common regulatory mechanism to mediate the body metabolism (e.g. energy, glucose and lipid homeostasis). Thus, a novel adiponectin-resistin (AR) index was proposed by taking into account both adiponectin and resistin levels to povide a better indicator of the metabolic homeostasis and metabolic disorders.

Established direct methods to quantify insulin sensitivity, such as euglycemic hyperinsulinemic clamp technique, are complex, troublesome, expensive, time-consuming, laborious and impractical in clinical practice. Surrogate indexes are available, but there are no universal cutoff points to define insulin resistance. Moreover, the existing surrogates indexes have low sensitivity and lack robustness for early diagnosis of insulin resistance in the general population. It is therefore of great interest to establish a convenient, cost-effective and reliable insulin sensitivity index. Thus, a novel insulin resistance (IR_AR_) index was proposed by integration of the AR index into an existing insulin resistance index to provide a more promising biomarker of insulin sensitivity for early diagnosis of T2DM and MS in the daily clinical practice and for large-scale clinical investigation. It also allows early treatment to prevent or to delay the onset of long-term severe complications including cardiovascular risk.

## Methods

### Subjects

All subjects were native to Malaysia and were males to avoid confounding effect of gender. The ages for all subjects were restricted to 40-70 years old because individuals with 40-70 years old contribute the majority cases of T2DM and MS. Subjects comprised three primary ethnic groups which were Malay, Chinese and Indian. Ethical clearance (reference number of Ethical Approval Letter was 612.17) to undertake this study was obtained from the University Malaya Medical Centre (UMMC) Ethics Committee. Informed consent was obtained from each subject, to whom possible consequences of the studies were explained. Each subject received a detailed questionnaire about the personal and family disease history and demographic data.

A case control study was designed. The subjects were classified into 208 controls, 174 MS without T2DM, 171 T2DM without MS, 256 T2DM with MS for a total 809 subjects. The controls were non-diabetic subjects who had no personal and family history and had no first degree relatives such as parent and sibling with T2DM and MS. The fasting plasma glucose levels for a control was in the normal range (<5.60 mmol/L) according to American Diabetes Association (ADA) 2003 diagnostic criteria [15]. Type 2 diabetes (T2DM) were identified as diabetic subjects who had fasting plasma glucose levels of ≥7.0 mmol/L and had been diagnosed by a diabetic physician with T2DM or had been taking diabetic medication.

Metabolic syndrome (MS) was defined according to International Diabetes Federation (IDF) 2005 diagnostic criteria [4]. According to the International Diabetes Federation (IDF) 2005 diagnostic criteria, for a person to be defined as having the MS they must have: central obesity (≥90 cm for South Asians male population), plus any two of the following four factors, which were raised triglycerides (≥1.70 mmol/L), reduced HDL cholesterol (<1.03 mmol/L in males), raised blood pressure (SBP ≥130 mmHg or DBP ≥85 mmHg or hypertension) and raised fasting plasma glucose (≥5.60 mmol/L or previously diagnosed T2DM).

The controls and subjects with T2DM or MS were selected from those attending the University Malaya Medical Centre (UMMC) for routine medical check-up or treatment. All subjects had not been diagnosed with other hereditary (e.g. cancer and cardiovascular disease) and infectious diseases (e.g. hepatitis) (Additional file 1, Supplementary Methods).

### Determination of anthropometric clinical and metabolic parameters

The metabolic parameters including fasting serum total adiponectin (Additional file 1, Figure S1A), resistin (Additional file 1, Figure S1B), insulin, total cholesterol, LDL cholesterol, HDL cholesterol, triglyceride, plasma glucose, and whole blood HbA1C levels were tested. The anthropometric parameters including the blood pressure, body mass index (BMI), waist circumference, and waist-to-hip ratio (WHR), were also measured or calculated. Surrogate indexes of insulin sensitivity including quantitative insulin sensitivity check index (QUICKI), homeostasis model assessment of insulin resistance (HOMA-IR) index, Bennett index, McAuley (1) index and McAuley (2) index were calculated (Additional file 1, Table S1).

### Statistical analysis

Significant differences in continuous variables among subject groups were confirmed by univariate analysis of covariance (ANCOVA) or multivariate analysis of covariance (MANCOVA) with PASW Statistics 18 Program (SPSS Inc, Chicago, Illinois, USA). General linear model was used, in which each subject group was included as a fixed factor. The models included ages as covariate. Type III sum-of-squares method was used. Then, 1,000 stratified bootstrap samples with bias corrected and accelerated (BCa) 95% confident interval were used for pair wise comparisons. Stratified bootstrapping method was used for stratified ethnicity and for multiple testing bias corrections due to deviation of normality. Spearman's rho rank correlation test was used to test the correlation between two variables. Nonparametric correlation test was used because most of the variables are not normally distributed. All p-values were two-tailed, and p-values below 0.05 were considered statistically significant.

## Results

### Clinical features of T2DM and MS subjects

The clinical characteristics of the controls, and subjects with type 2 diabetes (T2DM), and metabolic syndrome (MS) are shown in Table 1 and Table 2, which reflect the criteria used to define the subject groups. There was homogeneity for the covariate in terms of ages and ethnicity to match the case-control groups (Table 1). Subjects with T2DM and MS had higher (df = 3; F = 15.096; P = 1.45 × 10^-9^) serum insulin levels than the healthy subjects (Table 2). Serum insulin levels were the highest in subjects presenting with both T2DM and MS (Table 2).

**Table 1:** Anthropometric clinical parameters for each subject group

	Subjects				
		
Variables	Control (n = 208) group A	MS without T2DM (n = 174) group B	T2DM without MS (n = 171) group C	T2DM with MS (n = 256) group D	F-test; P-value (A vs. B vs. C vs. D)
Ethnics (Malay/Chinese/Indian)	75/73/60 C*	62/54/58 NS	43/62/66 A*, D**	101/75/80 C**	11.350; 0.0780
Ages (years)	55 (54, 56) D*	54 (53, 55) NS	55 (54, 56) D*	53 (52, 54) A*, C*	2.174; 0.0897
Diabetic medication (oral/injection)	NA	NA	157/14	197/59	NA
Duration of diabetes (years)	NA	NA	9 (8, 10)	8 (7, 9)	NA
Family history of diabetes (yes/no)	0/208	45/129	107/64	166/90	NA
Hypertension (yes/no)	0/208	123/51	13/158	219/37	NA
Dyslipidemia (yes/no)	0/208	174/0	0/171	244/12	NA
Smoking (yes/no)	39/169	48/126	55/116	91/165	NA
BMI (kg/m^2^)	24.39 (23.92, 24.87)	27.18 (26.69, 27.69)	24.13 (23.60, 24.67)	28.32 (27.84, 28.78)	66.309; 2.56 × 10^-38^
	B***, D***	A***, C***, D**	B***, D***	A***, B**, C***	
Waist (cm)	89 (88, 90)	96 (95, 97)	90 (88, 91)	100 (99, 102)	91.987; 3.49 × 10^-51^
	B***, D***	A***, C***, D***	B***, D***	A***, B***, C***	
WHR	0.898 (0.891, 0.904)	0.929 (0.921, 0.938)	0.914 (0.907, 0.922)	0.952 (0.946, 0.957)	50.414; 7.33 × 10^-30^
	B***, C***, D***	A***, C*, D***	A**, B*, D***	A***, B***, C***	
Systolic BP (mmHg)	133 (130, 135)	143 (140, 145)	129 (127, 131)	136 (134, 138)	21.188; 3.23 × 10^-13^
	B***, C*, D*	A***, C***, D***	A*, B***, D***	A*, B***, C***	
Diastolic BP (mmHg)	83 (81, 84)	88 (87, 90)	79 (78, 80)	83 (81, 84)	30.591; 9.87 × 10^-19^
	B***, C***	A***, C***, D***	A***, B***, D***	B***, C***	

Data are expressed as mean (95% confident interval). ANCOVA test was used, followed by pairwise comparison using 1,000 stratified bootstrap samples with bias corrected and accelerated (BCa) 95% CI for multiple testing bias corrections due to deviation of normality. The p-value was also adjusted for covariate ages with stratified ethnicity. The location of statistically significant differences are displayed as group's name (A, B, C, or D). Diabetic medication are including oral drugs (e.g. Metformin and Rosiglitazon) and/or injection of exogenous insulin. Significant levels: P* < 0.05, P** < 0.01, P*** < 0.001. Note: T2DM = type 2 diabetes; MS = metabolic syndrome; NA = not applicable; NS = not significant.

**Table 2:** Metabolic parameters for each subject group

	Subjects				
		
Variables	Control (n = 208) group A	MS without T2DM (n = 174) group B	T2DM without MS (n = 171) group C	T2DM with MS (n = 256) group D	F-test; P-value (A vs. B vs. C vs. D)
Total cholesterol (mmol/L)	5.03 (4.91, 5.14)	5.16 (5.03, 5.29)	4.68 (4.52, 4.85)	4.64 (4.50, 4.78)	12.412; 6.10 × 10^-8^
	C***, D***	C***, D***	A***, B***	A***, B***	
HDL cholesterol (mmol/L)	1.28 (1.25, 1.31)	1.07 (1.03, 1.10)	1.26 (1.22, 1.29)	1.04 (1.01, 1.07)	62.822; 1.70 × 10^-36^
	B***, D***	A***, C***	B***, D***	A***, C***	
LDL cholesterol (mmol/L)	3.14 (3.03, 3.26)	3.09 (2.95, 3.25)	2.88 (2.73, 3.04)	2.69 (2.57, 2.81)	10.656; 7.11 × 10^-7^
	C**, D***	C*, D***	A**, B*	A***, B***	
Triglyceride (mmol/L)	1.31 (1.25, 1.37)	2.20 (2.07, 2.34)	1.20 (1.12, 1.27)	2.02 (1.89, 2.16)	68.292; 2.40 × 10^-39^
	B***, C*, D***	A***, C***	A*, B***, D***	A***, C***	
Glucose (mmol/L)	5.13 (5.05, 5.23)	5.51 (5.39, 5.63)	8.04 (7.60, 8.50)	8.26 (7.94, 8.60)	118.021; 2.46 × 10^-63^
	B***, C***, D***	A***, C***, D***	A***, B***	A***, B***	
HbA1C (%)	5.63 (5.57, 5.69)	5.82 (5.76, 5.89)	8.07 (7.78, 8.36)	8.09 (7.90, 8.28)	225.382; 4.32 × 10^-106^
	B***, C***, D***	A***, C***, D***	A***, B***	A***, B***	
Insulin (μU/mL)	10.99 (10.05, 11.97)	19.94 (16.95, 23.42)	14.86 (12.76, 17.04)	24.32 (20.85, 27.95)	15.096; 1.45 × 10^-9^
	B***, C**, D***	A***, C*	A**, B*, D**	A***, C**	

Data are expressed as mean (95% confident interval). ANCOVA test was used, followed by pairwise comparison using 1,000 stratified bootstrap samples with bias corrected and accelerated (BCa) 95% CI for multiple testing bias corrections due to deviation of normality. The p-value was also adjusted for covariate ages with stratified ethnicity. The location of statistically significant differences are displayed as group's name (A, B, C, or D). Significant levels: P* < 0.05, P** < 0.01, P*** < 0.001. Note: T2DM = type 2 diabetes; MS = metabolic syndrome.

### Serum adiponectin levels

**Figure 1:**
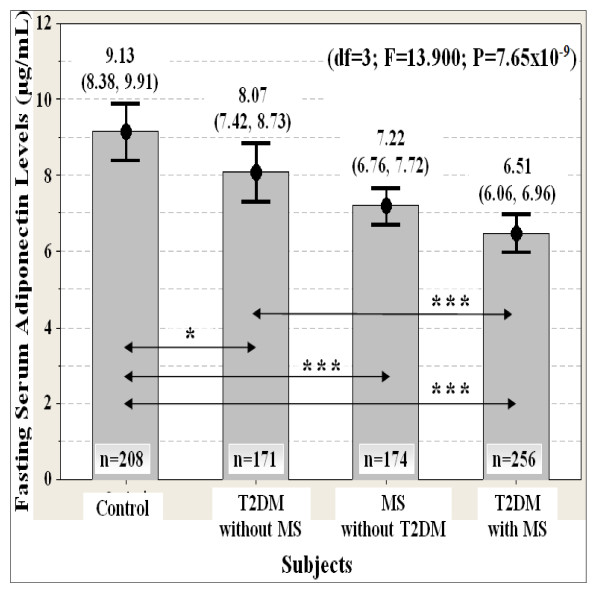
**Association of fasting serum adiponectin levels with T2DM and MS susceptibility (n = 809)**. Data are expressed as mean (95% confident interval). ANCOVA test was used, followed by pairwise comparison using 1,000 stratified bootstrap samples with bias corrected and accelerated (BCa) 95% CI for multiple testing bias corrections due to deviation of normality. The p-value was also adjusted for covariate ages with stratified ethnicity. The location of statistically significant differences are displayed as the double arrow. Significant levels: P* < 0.05, P** < 0.01, P*** < 0.001. Note: T2DM = type 2 diabetes; MS = metabolic syndrome.

Hypoadiponectinemia was strongly associated (df = 3; F = 13.900; P = 7.65 × 10^-9^) with increased risk of type 2 diabetes (T2DM) and metabolic syndrome (MS) in Malaysian men (Figure 1). Serum adiponectin levels were significantly lower in MS subjects who do not yet manifest T2DM as compared to the healthy subjects (Figure 1). Serum adiponectin levels were also lower in T2DM subjects who do not yet manifest MS as compared to the healthy subjects (Figure 1). Interestingly, serum adiponectin levels were further down-regulated in subjects presenting with both T2DM and MS (Figure 1). These findings were consistent with previous reports on adiponectin in most epidemiological studies [5,8,16] and meta-analyses [9].

Serum adiponectin levels were positively correlated with serum HDL cholesterol levels, QUICKI, Bennett, McAuley (1) and McAuley (2) indexes, and it was negatively correlated with BMI, waist, WHR, HOMA-IR index, serum triglyceride, insulin, resistin, plasma glucose and whole blood HbA1C levels (Table 3 and Table 4). The strongest correlation of serum adiponectin levels was with the insulin resistance indexes, serum HDL cholesterol, triglyceride and insulin levels (Table 3 and Table 4). Taking these findings together, it showed that adiponectin plays an important role in the modulation of lipid homeostasis (e.g. fatty acids oxidation) and insulin sensitivity. Adiponectin may also involve in the mediation of glucose homeostasis.

**Table 3:** Correlation of the anthropometric clinical and metabolic parameters

Variables	Adiponectin (μg/mL) (n = 809)	Resistin (ng/mL) (n = 809)	AR Index (n = 809)
Ages (years)	r = +0.1347	r = +0.0422	r = -0.0380
	P = 1.22 × 10^-4^***	P = 0.2299	P = 0.2794
BMI (kg/m^2^)	r = -0.1480	r = + 0.1266	r = + 0.1753
	P = 2.38 × 10^-5 ^***	P = 3.08 × 10^-4 ^***	P = 5.27 × 10^-7 ^***
Waist (cm)	r = -0.2126	r = +0.2104	r = +0.2694
	P = 1.01 × 10^-9 ^***	P = 1.50 × 10^-9 ^***	P = 6.35 × 10^-15^***
WHR	r = -0.1765	r = + 0.1911	r = + 0.2370
	P = 4.39 × 10^-7 ^***	P = 4.33 × 10^-8 ^***	P = 8.67 × 10^-12 ^***
Systolic BP (mmHg)	r = + 0.0323	r = + 0.0564	r = + 0.0170
	P = 0.3586	P = 0.1088	P = 0.6285
Diastolic BP (mmHg)	r = + 0.0355	r = -0.0398	r = -0.0591
	P = 0.3131	P = 0.2587	P = 0.0931
Total cholesterol (mmol/L)	r = +0.0696	r = -0.0648	r = -0.0947
	P = 0.0478 *	P = 0.0654	P = 0.0070 **
HDL cholesterol (mmol/L)	r = +0.3341	r = -0.1065	r = -0.2658
	P = 1.53 × 10^-22 ^***	P = 0.0024 **	P = 1.50 × 10^-14 ^***
LDL cholesterol (mmol/L)	r = +0.0618	r = -0.0610	r = -0.0849
	P = 0.0791	P = 0.0829	P = 0.0157 *
Triglyceride (mmol/L)	r = -0.2072	r = +0.0358	r = +0.1349
	P = 2.70 × 10^-9 ^***	P = 0.3093	P = 1.18 × 10^-4 ^***
Glucose (mmol/L)	r = -0.1725	r = +0.3077	r = +0.3279
	P = 8.00 × 10^-7 ^***	P = 3.36 × 10^-19 ^***	P = 9.81 × 10^-22 ^***
HbA1C (%)	r = -0.2254	r = + 0.3291	r = + 0.3716
	P = 8.97 × 10^-11 ^***	P = 6.84 × 10^-22 ^***	P = 6.82 × 10^-28 ^***
Insulin (μU/mL)	r = -0.2374	r = + 0.1598	r = + 0.2395
	P = 8.00 × 10^-12 ^***	P = 4.94 × 10^-6 ^**	P = 5.11 × 10^-12 ^***
Adiponectin (μg/mL)	NA	r = -0.1053	NA
	NA	P = 0.0027 **	NA
Resistin (ng/mL)	r = -0.1053	NA	NA
	P = 0.0027 **	NA	NA

Spearman's rho rank correlation test was used because most of the variables are not normally distributed. Significant levels: P* < 0.05, P** < 0.01, P*** < 0.001. Note: r = Spearman's rho rank correlation coefficient; BMI = body mass index; WHR = waist-to-hip ratio; BP = blood pressure; AR = adiponectin-resistin; NA = not applicable.

**Table 4:** Correlation of the insulin resistance indexes

Insulin resistance indexes	Adiponectin (μg/mL) (n = 809)	Resistin (ng/mL) (n = 809)	AR index (n = 809)
HOMA-IR index	r = -0.2639	r = +0.2615	r = +0.3355
	P = 2.37 × 10^-14 ^***	P = 4.04 × 10^-14 ^***	P = 9.73 × 10^-23 ^***
QUICKI	r = +0.2736	r = -0.2844	r = -0.3530
	P = 1.41 × 10^-16 ^***	P = 1.63 × 10^-16 ^***	P = 3.71 × 10^-25 ^***
Bennett index	r = +0.2690	r = -0.2704	r = -0.3463
	P = 7.36 × 10^-15 ^***	P = 5.27 × 10^-15 ^***	P = 3.57 × 10^-24 ^***
McAuley (1) index	r = +0.2824	r = -0.1253	r = -0.2408
	P = 2.67 × 10^-16 ^***	P = 3.54 × 10^-4 ^***	P = 3.92 × 10^-12 ^***
McAuley (2) index	r = +0.2799	r = -0.1355	r = -0.2481
	P = 5.03 × 10^-16 ^***	P = 1.11 × 10^-4 ^***	P = 8.14 × 10^-13 ^***

Spearman's rho rank correlation test was used. Significant levels: P* < 0.05, P** < 0.01, P*** < 0.001. Note: r = Spearman's rho rank correlation coefficient; HOMA-IR = homeostasis model assessment of insulin resistance; QUICKI = quantitative insulin sensitivity check index; AR = adiponectin-resistin.

### Serum resistin levels

**Figure 2:**
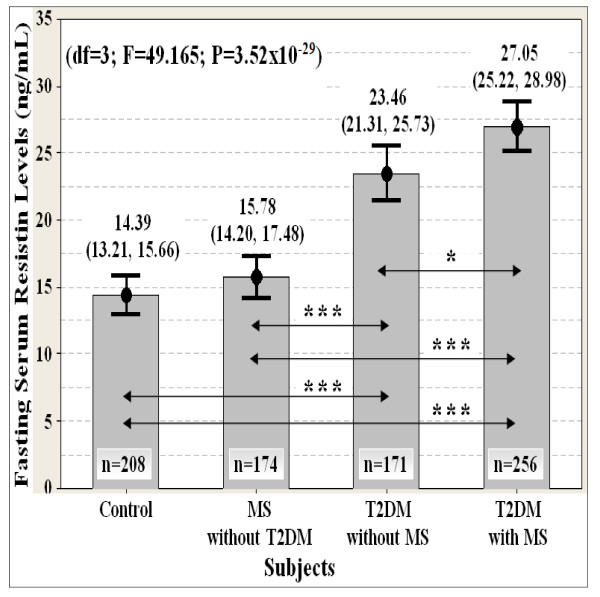
**Association of fasting serum resistin levels with T2DM and MS susceptibility (n = 809)**. Data are expressed as mean (95% confident interval). ANCOVA test was used, followed by pairwise comparison using 1,000 stratified bootstrap samples with bias corrected and accelerated (BCa) 95% CI for multiple testing bias corrections due to deviation of normality. The p-value was also adjusted for covariate ages with stratified ethnicity. The location of statistically significant differences are displayed as the double arrow. Significant levels: P* < 0.05, P** < 0.01, P*** < 0.001. Note: T2DM = type 2 diabetes; MS = metabolic syndrome.

Hyperresistinemia was strongly associated (df = 3; F = 49.165; P = 3.52 × 10^-29^) with increased risk of type 2 diabetes (T2DM) and metabolic syndrome (MS) in Malaysian men (Figure 2). Serum resistin levels were significantly higher in T2DM subjects who do not yet manifest MS as compared to the healthy subjects (Figure 2). However, there was no significant difference in serum resistin levels between the healthy and MS subjects who do not yet manifest T2DM (Figure 2). Hyperresistinemia was more severe in subjects presenting with both T2DM and MS (Figure 2). These findings were consistent with previous reports on resistin in most epidemiological studies [6,11].

Serum resistin levels were positively correlated with BMI, waist, WHR, HOMA-IR index, serum insulin, plasma glucose and whole blood HbA1C levels, and it was negatively correlated with serum HDL cholesterol and adiponectin levels, QUICKI, Bennett, McAuley (1) and McAuley (2) indexes (Table 3 and Table 4). Serum resistin levels had the strongest correlation with the insulin resistance indexes, plasma glucose and whole blood HbA1C levels (Table 3 and Table 4). Taking these findings together, it showed that resistin plays an important role in the regulation of glucose homeostasis (e.g. gluconeogenesis) and insulin sensitivity.

### Adiponectin-resistin interaction

The interaction effect of adiponectin and resistin was more strongly associated (P≤2.32x10^-34^) with increased risk of type 2 diabetes (T2DM) and metabolic syndrome (MS) compared to hypoadiponectinemia (P = 7.65 × 10^-9^) and hyperresistinemia (P = 3.52 × 10^-29^) alone (Table 5, Figure 1 and Figure 2). The condition of hypoadiponectinemia and hyperresistinemia tend to concurrent in subjects presenting with both T2DM and MS (Figure 1 and Figure 2). Also, serum adiponectin levels were negatively correlated (P = 0.0027) with serum resistin levels (Table 3). Given the opposite effects of adiponectin and resistin on the insulin sensitivity, it speculates that relative proportion of adiponectin-to-resistin might potentially influence the risk of T2DM and MS (Table 3 and Table 4). Taking these findings together, it may be speculated that adiponectin and resistin interact to modulate metabolic homeostasis.

**Table 5:** Multivariate analysis for adiponecin-resistin interaction

Multivariate test	Value	F	Hypothesis df	Error df	P-value
Pillai's trace	0.201	29.920	6	1608	3.70 × 10^-34 ^***
Wilks' lambda	0.803	31.050	6	1606	1.90 × 10^-35 ^***
Hotelling's trace	0.241	32.182	6	1604	9.76 × 10^-37 ^***
Roy's largest root	0.219	58.779	3	804	2.32 × 10^-34 ^***

MANCOVA test with 1,000 stratified bootstrap samples, and bias corrected and accelerated (BCa) 95% CI was used for multiple testing bias corrections due to deviation of normality. The p-value was also adjusted for covariate ages with stratified ethnicity. The model included adiponectin and resistin levels as dependent variables, subjects as fixed factors, ages and ethnicity as covariates. Subjects were comprising of 208 controls, 171 T2DM without MS, 174 MS without T2DM, and 256 T2DM with MS. Significant levels: P* < 0.05, P** < 0.01, P*** < 0.001. Note: T2DM = type 2 diabetes; MS = metabolic syndrome.

### Formulation of the adiponectin-resistin (AR) index

Taking the findings together, it may be speculated that the integration of adiponectin and resistin in a novel unified index would be better reflected metabolic homeostasis and metabolic disorders. Adiponectin (A_0_) and resistin (R_0_) levels having diametrically opposed physiological effects in the present study (Table 3, Table 4, Figure 1 and Figure 2). Thus, A_0 _and R_0 _are unified by multiplicative inverse as follows

(1) $$\alpha =\left(\text{1}/{\text{A}}_{0}\right)\times {\text{R}}_{0}={\text{R}}_{0}/{\text{A}}_{0}$$

Then (1) is logarithmically transformed for normalization,

(2) $$\begin{array}{c} \beta ={\text{log}}_{10}(\alpha )={\text{log}}_{10}({\text{R}}_{0}/{\text{A}}_{0})\\ ={\text{log}}_{10}({\text{R}}_{0})-{\text{log}}_{10}({\text{A}}_{0}) \end{array}$$

Lastly, a numerical constant 1 is added to (2) to get a positive integer of the AR index

(3a) $$\begin{array}{c} \text{AR index}=\text{1}+\beta \\ =\text{1}+{\text{log}}_{\text{1}0}\left({\text{R}}_{0}\right)-{\text{log}}_{\text{1}0}\left({\text{A}}_{0}\right) \end{array}$$

Note: R_0 _= fasting serum total resistin levels in ng/mL;

A_0 _= fasting serum total adiponectin levels in μg/mL.

### Evaluation of the adiponectin-resistin (AR) index

**Figure 3:**
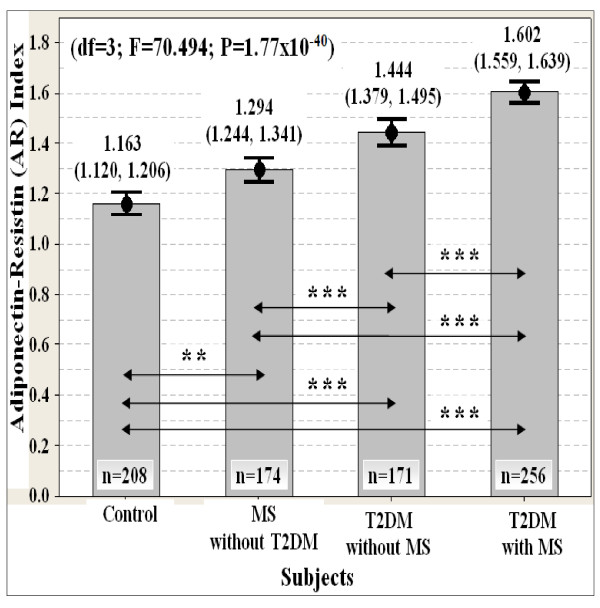
**Association of the adiponectin-resistin (AR) index with T2DM and MS susceptibility (n = 809)**. Data are expressed as mean (95% confident interval). ANCOVA test was used, followed by pairwise comparison using 1,000 stratified bootstrap samples with bias corrected and accelerated (BCa) 95% CI for multiple testing bias corrections due to deviation of normality. The p-value was also adjusted for covariate ages with stratified ethnicity. The location of statistically significant differences are displayed as the double arrow. Significant levels: P* < 0.05, P** < 0.01, P*** < 0.001. Note: T2DM = type 2 diabetes; MS = metabolic syndrome.

The adiponectin-resistin (AR) index was more strongly associated (df = 3; F = 70.494; P = 1.77 × 10^-40^) with increased risk of type 2 diabetes (T2DM) and metabolic syndrome (MS) than adiponectin (df = 3; F = 13.900; P = 7.65 × 10^-9^) and resistin (df = 3; F = 49.165; P = 3.52 × 10^-29^) levels alone (Figure 1, Figure 2 and Figure 3). The AR index was the lowest in controls, followed by the subjects with MS and T2DM (Figure 3). The AR index was the highest in subjects presenting with both T2DM and MS (Figure 3).

The normal reference range of the AR index for healthy individuals was between 1.120 and 1.206 (Figure 3). The minimum cut-off values of the novel AR index for diagnosis of T2DM and MS in Malaysian men were between 1.206 and 1.244 (Figure 3). An individual whose AR index is 1.244 or greater (indicator for developing MS) is defined as being in a metabolic syndrome or pre-diabetic state (Figure 3). When the AR index is 1.379 or greater (indicator for developing T2DM), the individual is diagnosed as having type 2 diabetes (Figure 3). When the AR index is 1.559 or greater (indicator for developing of diabetic complications), the individual is diagnosed as having both type 2 diabetes and metabolic syndrome (Figure 3). These predictive values of the AR index were only applicable to Malaysian men with 95% confident interval (Figure 3).

The AR index was correlated with a higher number of MS components than adiponectin and resistin levels alone (Table 3). The AR index was also more strongly correlated with the insulin resistance indexes and other risk factors including serum insulin, plasma glucose and whole blood HbA1C levels than adiponectin and resistin levels alone (Table 3 and Table 4). Thus, the AR index may play a greater role in reflecting circulating metabolite levels and insulin sensitivity than adiponectin and resistin levels alone.

### Formulation of the insulin resistance (IR_AR_) index

Among the existing insulin resistance indexes, quantitative insulin sensitivity check index (QUICKI) had the stongest correlation (P = 3.71 × 10^-25^) with the adiponectin-resistin (AR) index (Table 4). The QUICKI and AR indexes are formulated as follows

(3b) $$\text{AR index}=\text{1}+{\text{log}}_{\text{1}0}\left({\text{R}}_{0}\right)-{\text{log}}_{\text{1}0}\left({\text{A}}_{0}\right)\;$$

(4) $$\text{QUICKI}=\text{1}/\left[{\text{log}}_{\text{1}0}\left({\text{I}}_{0}\right){+\text{log}}_{\text{1}0}\left({\text{G}}_{0}\right)\right]\;$$

The QUICKI was negatively correlated with the AR index (Table 4). Therefore, (3) and (4) are unified by multiplicative inverse as follows

(5) $$\begin{array}{c} {\text{IR}}_{\text{AR}}\text{ index }=\left(\text{AR index}\right)/\left(\text{QUICKI}\right)\\ =\left(\text{1}/\text{QUICKI}\right)\times \left(\text{AR index}\right)\\ =\left[{\text{log}}_{\text{1}0}\left({\text{I}}_{0}\right)+{\text{log}}_{\text{1}0}\left({\text{G}}_{0}\right)\right]\times \left[\text{1}+{\text{log}}_{\text{1}0}\left({\text{R}}_{0}\right)-{\text{log}}_{\text{1}0}\left({\text{A}}_{0}\right)\right]\\ ={\text{log}}_{\text{1}0}\left({\text{I}}_{0}{\text{G}}_{0}\right)\;\;\text{​}\left[\text{1}+{\text{log}}_{\text{1}0}\left({\text{R}}_{0}/{\text{A}}_{0}\right)\right] \end{array}$$

Lastly, (5) is simplify to become a finalized IR_AR _index as follows

(6) $${\text{IR}}_{\text{AR}}\text{ index}={\text{log}}_{\text{1}0}\left({\text{I}}_{0}{\text{G}}_{0}\right)+{\text{log}}_{\text{1}0}\left({\text{I}}_{0}{\text{G}}_{0}\right){\text{ log}}_{\text{1}0}\left({\text{R}}_{0}/{\text{A}}_{0}\right)$$

Note: I_0 _= fasting serum insulin levels in μU/mL;

G_0 _= fasting plasma glucose levels in mg/dL;

R_0 _= fasting serum total resistin levels in ng/mL;

A_0 _= fasting serum total adiponectin levels in μg/mL.

### Evaluation of the insulin resistance (IR_AR_) index

**Figure 4:**
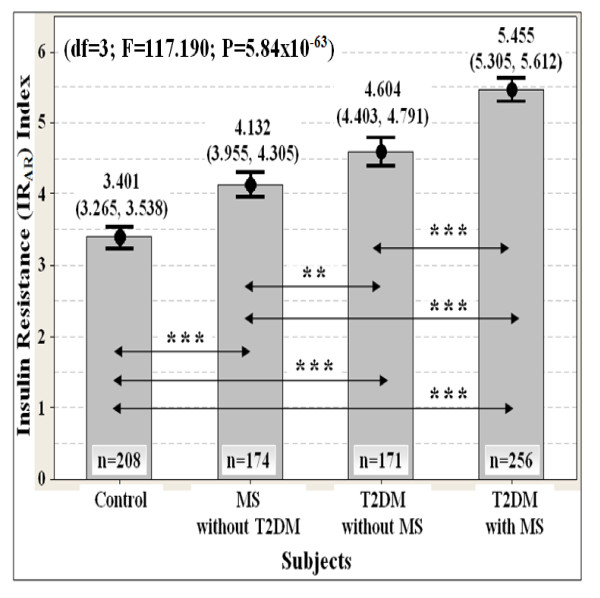
**Evaluation of insulin sensitivity with the insulin resistance (IR_AR_) index (n = 809)**. Data are expressed as mean (95% confident interval). ANCOVA test was used, followed by pairwise comparison using 1,000 stratified bootstrap samples with bias corrected and accelerated (BCa) 95% CI for multiple testing bias corrections due to deviation of normality. The p-value was also adjusted for covariate ages with stratified ethnicity. The location of statistically significant differences are displayed as the double arrow. Significant levels: P* < 0.05, P** < 0.01, P*** < 0.001. Note: T2DM = type 2 diabetes; MS = metabolic syndrome.

The insulin resistance (IR_AR_) index (df = 3; F = 117.190; P = 5.84 × 10^-63^) may better predict insulin resistance than classical surrogate indexes including the QUICKI (df = 3; F = 103.892; P = 7.62 × 10^-57^) (Table 6). The IR_AR _index was higher in the MS subjects who do not yet manifest T2DM as compared to the healthy subjects (Figure 4). The IR_AR _index was also higher in the T2DM subjects who do not yet manifest MS as compared to the healthy subjects (Figure 4). The IR_AR _index was the highest in subjects presenting with both T2DM and MS (Figure 4). In addition, the IR_AR _index had high precision, consistency and reproducibility in the assessment of the insulin resistance (Figure 4).

**Table 6:** Evaluation of insulin sensitivity with the insulin resistance indexes

	Subjects				
		
Insulin resistance indexes	Control (n = 208) group A	MS without T2DM (n = 174) group B	T2DM without MS (n = 171) group C	T2DM with MS (n = 256) group D	F-test; P-value (A vs. B vs. C vs. D)
HOMA-IR index	2.546 (2.317, 2.779) )	5.038 (4.334, 5.923)	5.066 (4.367, 5.968)	8.706 (7.476, 10.183)	26.447; 2.55 × 10^-16^
	B***, C***, D***	A***, D***	A***, D***	A***, B***, C***	
QUICKI	0.346 (0.342, 0.351)	0.317 (0.313, 0.320)	0.319 (0.314, 0.324)	0.296 (0.293, 0.299)	103.892; 7.62 × 10^-57^
	B***, C***, D***	A***, D***	A***, D***	A***, B***, C***	
Bennett index	0.309 (0.293, 0.327)	0.228 (0.220, 0.237)	0.240 (0.224, 0.257)	0.181 (0.175, 0.186)	76.614; 1.38 × 10^-43^
	B***, C***, D***	A***, D***	A***, D***	A***, B***, C***	
McAuley (1) index	7.235 (7.009, 7.474)	5.305 (5.151, 5.475)	7.095 (6.845, 7.375)	5.347 (5.187, 5.518)	105.878; 9.02 × 10^-58^
	B***, D***	A***, C***	B***, D***	A***, C***	
McAuley (2) index	7.447 (7.227, 7.664)	5.496 (5.326, 5.651)	7.331 (7.103, 7.580)	5.486 (5.336, 5.627)	120.944; 1.20 × 10^-64^
	B***, D***	A***, C***	B***, D***	A***, C***	
IR_AR _index	3.401 (3.265, 3.538)	4.132 (3.955, 4.305)	4.604 (4.403, 4.791)	5.455 (5.305, 5.612)	117.190; 5.84 × 10^-63^
	B***, C***, D***	A***, C**, D***	A***, B**, D***	A***, B***, C***	

Data are expressed as mean (95% confident interval). ANCOVA test was used, followed by pairwise comparison using 1,000 stratified bootstrap samples with bias corrected and accelerated (BCa) 95% CI for multiple testing bias corrections due to deviation of normality. The p-value was also adjusted for covariate ages with stratified ethnicity. The location of statistically significant differences are displayed as group's name (A, B, C, or D). Significant levels: P* < 0.05, P** < 0.01, P*** < 0.001. Note: T2DM = type 2 diabetes; MS = metabolic syndrome; HOMA-IR = homeostasis model assessment of insulin resistance; QUICKI = quantitative insulin sensitivity check index, IR_AR _= insulin resistance (adiponectin-resistin).

The normal reference range of the IR_AR _index for insulin sensitive individuals was between 3.265 and 3.538 (Figure 4). The minimum cut-off values of the novel IR_AR _index for insulin resistance assessment in Malaysian men were between 3.538 and 3.955 (Figure 4). An individual whose IR_AR _index is between 3.955 and 4.305 (indicator for developing MS) is defined as being in a mild insulin resistance state (Figure 4). An individual whose IR_AR _index is between 4.403 and 4.791 (indicator for developing T2DM) is defined as being in a moderate insulin resistance state (Figure 4). An individual whose IR_AR _index is between 5.305 and 5.612 (indicator for developing diabetic complications) is defined as being in a severe insulin resistance state (Figure 4). These predictive values of the IR_AR _index were only applicable to Malaysian men with 95% confident interval (Figure 4).

## Discussion

### Hypoadiponectinemia

Circulating levels of adiponectin are highly heritable, with more than 30-70% of its variability explained by genetics factors [17,18]. A recent comprehensive linkage disequilibrium mapping revealed many SNPs in the adiponectin gene (*ADIPOQ*) were strongly associated with circulating adiponectin levels [18]. In addition, an extensive bioinformatics analysis revealed the *ADIPOQ *region might be a high copy number variable region which potentially influences circulating adiponectin levels [18]. Furthermore, *ADIPOQ *is a major gene influencing circulating adiponectin levels from the genome-wide perspective [18,19]. The area of chromosome 3q27 where the *ADIPOQ *gene is located has been identified by genome-wide linkage studies (GWLSs) to be a susceptibility locus for risk for the type 2 diabetes (T2DM) [20], metabolic syndrome (MS) [21] and cardiovascular disease [22,23]. However, this was not shown in recently published meta-analyses of genome-wide association studies (GWASs) [24-27]. The *ADIPOQ *gene is located nearby the *IGF2BP2 *(insulin-like growth factor 2 mRNA-binding protein 2) and *AOMS1 *(abdominal obesity-metabolic syndrome QTL 1) genes on chromosome 3q27 (based on Ensembl database). The other T2DM and MS susceptibility genes including *HDLCQ5 *(high density lipoprotein cholesterol level QTL 5) (on chromosome 3q24-q26), *AGTR1 *(angiotension receptor 1) (on chromosome 3q24), *FGQTL6 *(fasting plasma glucose level QTL 6) (on chromosome 3q21), *RETNLB *(resistin-like beta) (on chromosome 3q13.13), *HYT7 *(hypertension, essential, susceptibility to, 7) (on chromosome 3p14.1-q12.3), *PPARG *(peroxisome proliferator activated receptor gamma) (on chromosome 3p25.2) and *ABHD5 *(abhydrolase domain containing 5) (on chromosome 3p25.3-p24.3) may also in linkage disequilibrium with the *ADIPOQ *gene (based on Ensembl database). Thus, these genes may influence the functional mechanism and expression of the *ADIPOQ *gene including adiponectin levels.

Adiponectin has already been identified as a potential target for therapeutics to treat T2DM [28] and MS [29-31] in a series of clinical trials. Many existing drugs have been found to increase adiponectin levels, including statins (e.g. pravastatin, simvastatin, rosuvastatin and atorvastatin), angiotensin converting enzyme inhibitors and angiotensin receptor blockers (e.g. ramipril, quinapril, telmisartan, irbesartan and candesartan), β-adrenergic agonists, and thiazolidinediones (e.g. pioglitazone and rosiglitazone) [32]. Other drugs that increase serum adiponectin levels were including non-statin anti-hyperlipidemic drugs (e.g. fenofibrate), non-TZD anti-diabetic drugs (e.g. acarbose and sulfonylurea glimepiride) and androgen blockers [32]. A meta-analysis including 19 prospective studies had pointed out an increase in serum adiponectin levels in subjects undergoing treatment with thiazolidinediones (TZD) [33]. Moreover, a systematic review including 33 clinical trials showed that exercise of varying prescription was able to increase serum adiponectin levels [34]. However, a recent survey revealed the paradoxical findings regarding the role of adiponectin in human disease [35]. According to the concept of the reversal epidemiology in the adiponectin physiology, adiponectin would behave as an insulin sensitizing and cardioprotective factor in the health state and as a wasting marker in the advanced states of disease [35].

### Hyperresistinemia

Genetic variants in *RETN *(the resistin gene) have been examined by many groups, and it was estimated that up to 70% of the variation in circulating resistin levels could be explained by genetic factors [36]. Moreover, recent fine-mapping of SNP studies which covering the full *RETN *gene revealed several SNPs of the *RETN *gene account for the high variablity of resistin levels [37]. In the San Antonio Family Heart Study, the maximum linkage signal for the *RETN *expression was found on chromosome 19p13 (location of the *RETN *gene) [38]. In addition, the *RETN *gene is located nearby the *INSR *(insulin receptor) and *LDLR *(low density lipoprotein receptor) genes on chromosome 19p13.2 (based on Ensembl database). This suggests that *RETN *expression may be cis-regulated, meaning there are variants in or near the *RETN *gene that influence the abundance of its mRNA [38]. The other T2DM and MS susceptibility genes including *ATHS *(atherosclerosis susceptibility, lipoprotein associated) (on chromosome 19p13.3-p13.2), *AKT2 *(murine thymoma viral homolog-2) (on chromosome 19q13.2), *FFAR 1 *(free fatty acids receptor 1) (on chromosome 19q13.12), *FFAR 2 *(on chromosome 19q13.12) and *FFAR 3 *(on chromosome 19q13.12) may also in linkage disequilibrium with the *RETN *gene (based on Ensembl database). In the meta-analyses of genome-wide linkage studies (GWLSs), suggestive evidence of linkage was observed for LDL cholesterol [39,40], apolipoprotein B [39], total cholesterol [40], and HDL cholesterol [40] on chromosome 19p13. These provide compelling evidence that the region of chromosome 19p13 harbor important determinants of lipid levels in individuals with T2DM [40]. However, the *RETN *gene was not detected as a susceptibility locus for risk for the T2DM, MS and cardiovascular disease in recently published meta-analyses of genome-wide association studies (GWASs) [24-27]. Thus, whether the *RETN *gene modulates metabolic homeostasis independently or functions in concert with other causative genes in a haplotype block remains to be elucidated.

Compared to adiponectin, the effects of drugs treatment on resistin levels in patients with T2DM and MS is less described. However, a few clinical trials showed that the anti-diabetic (e.g. rosiglitazone) [41], anti-hypertensive (e.g. amlodipine) [31] and anti-dyslipidemic (e.g. pitavastatin) [42] drugs were able to reduce circulating resistin levels and may contribute to improving insulin action in patients with T2DM and MS. Recently, Koh et al. reported that amlodipine (a calcium channel blocker) therapies significantly decreased resistin levels greater than ramipril (an angiotensin-converting enzyme inhibitor) or candesartan (an angiotensin II receptor antagonist) therapies in patients with hypertension [31]. In addition, resistin concentration decreased after long-term exercise training in overweight adolescents [43]. A recent long-term follow-up study revealed elevated serum resistin levels were associated with higher rates of mortality and hospitalization for heart failure [11]. However, serum resistin levels do not add prognostic information among high-risk persons with established coronary heart disease [11].

### Adiponectin-resistin interaction

In consistent with our study, a significant inverse correlation between serum adiponectin and resistin levels has also been reported in the literatures [12,13]. It has been reported that those with highest increases of adiponectin also displayed a trend towards a decline in resistin levels [13]. Tuttolomondo et al. demonstrated that diabetic subjects with diabetic foot had higher resistin levels and lower adiponectin levels compared to diabetics without diabetic foot [44]. Both hypoadiponectinemia and hyperresistinemia were also positively correlated with diabetes duration, hypertension, dyslipidemia, retinopathy, previous cerebrovascular disease (TIA/ischemic stroke), neuropathy, and diabetic foot grade [44]. Furthermore, both hypoadiponectinemia and hyperresistinemia were associated with out-of-clinic hypertension [45] and may have prognostic significance for future cardiovascular events in patients with masked hypertension [46]. Elevated resistin opposed to adiponectin plasma levels was proposed to be a strong predictive factor for the occurrence of major adverse cardiac events in patients with stable multivessel coronary artery disease over 1-year follow-up [47]. A recent prospective longitudinal pilot trial revealed systemic therapy ameliorates endothelial cell function by increase adiponectin and decrease resistin levels in patients with plaque-type psoriasis [48]. In addition, analysis with (18)F-fluorodeoxyglucose positron emission tomography revealed both adiponectin and resistin may be useful as biomarkers to reflect vascular inflammation [49]. Thus, the balance of the opposite effects of adiponectin and resistin at the level of the endothelial cell may be an important determinant of endothelial dysfunction, and in turn the progress of atherosclerosis.

Several studies have illustrated the interaction between adipokines (including adiponectin and resistin) and adenosine 5' monophosphate-activated protein kinase (AMPK), and highlighted AMPK as a potential target for the development of tissue-specific AMPK modulators in the treatment of T2DM and MS [50]. In patients with metabolic syndrome, thiazolidinediones (TZDs) including pioglitazone [51] and rosiglitazone [52] treatment markedly increased adiponectin and decreased resistin levels. These treatment effects on both adiponectin and resistin may further contribute to the AMPK activation exerted by TZDs [52]. A recent randomized double blind clinical trial demonstrated that short-term treatment with losartan (an angiotensin II receptor antagonist drug) improved both adiponectin and resistin levels in hypertensive subjects [53]. Furthermore, fenofibrate therapy improved both adiponectin and resistin levels, and may directly contribute to improving insulin sensitivity in hypertriglyceridemic patients [54]. These may in turn exert detrimental and beneficial effects on glucose and lipid metabolism.

Crosstalk of adipokines including adiponectin and resistin at the expression level and/or sites of brain's central action may eventually lead to the development and perpetuation of T2DM and MS [55]. The intricate interactions between adiponectin and resistin with catecholamines may play an integral role in metabolism [56]. In addition, adipocyte-derived microvesicles mediated transport of adiponectin and resistin gene transcripts into macrophages and might play a role as a novel intercellular communication tool by transporting RNA in paracrine and possibly endocrine manners [57]. Miyamoto et al. found that resistin may increase the susceptibility of metabolic syndrome by modulating adiponectin secretion from adipocytes [58]. Resistin may enhance hepatic gluconeogenesis, presumably by antagonizing adiponectin, which inhibits enzymes involved in gluconeogenesis through AMPK activation [58]. Furthermore, SNP-420C > G of the resistin gene was associated with lower circulating adiponectin levels in a Japanese cohort study [58]. Thus, the transcriptional activity of the resistin gene may also influence circulating adiponectin levels.

Adiponectin [7] and resistin [10] hormones are considered significant root factors for the regulation of energy, glucose, and lipid homeostasis as well as insulin signalling pathway. Moreover, it has been reported that the overall structure of multimeric assembly of the resistin is similar to that of adiponectin [14]. Both have been characterized as coiled-coil trimers that formed tail-to-tail hexamers through disulfide bonds near their amino termini [14]. Furthermore, both of these hormones circulate in serum in two distinct assembly states [14]. The comparable domain architecture of these two adipocyte-specific hormones, despite having diametrically opposed physiological effects, suggested a common regulatory mechanism in metabolic homeostasis [14].

### The adiponectin-resistin (AR) index

It has been reported that the adiponectin-resistin ratio might be potentially useful in prediction of the future cardiovascular risk in women with the polycystic ovary syndrome [13]. Moreover, changes of the relative proportion of adiponectin to resistin might play a more important role in hormonal disturbances in polycystic ovary syndrome than the absolute concentrations of these adipokines [59]. In addition, mice under chronic variable stress and fed with a high-fat diet showed impaired glucose tolerance associated with low plasma adiponectin-resistin ratios [60]. It seems that changes of circulating adiponectin and resistin levels may be the effect of their mutual interaction in adipose tissue. Thus, the AR index that included information on both serum adiponectin and resistin levels may has a more integrated and concentrated explanation than single measure of serum adiponectin and resistin levels in the present study.

Taking these studies together, adiponectin and resistin may be useful markers for insulin resistance and the variables that can integrate the abnormalities of the metabolic syndrome and cardiometabolic function. For this reason we attempted to estimate a threshold for the AR index for the identification of T2DM and MS. Although further studies may be necessary to confirm the efficacy of periodically measuring AR index in the management of insulin resistance, MS and T2DM, our study certainly highlights the potential for the AR index to move one step closer to becoming an established biomarker of the metabolic status. The evaluation of intervention strategies can be facilitated and strengthened by the use of the AR index that measure biological parameters of disease progression and therapeutic response. Routine assessment of the AR index may allow for a better understanding of the underlying disease conditions and optimization of anti-diabetic therapy targeting beyond simple glycemic control. Thus, the AR index has a potential for routinely available in general clinical practice and make a meaningful contribution to patient care.

### The insulin resistance (IR_AR_) index

Given the complicated nature of the euglycemic hyperinsulinemic clamp technique and the potential dangers of hypoglycemia in some patients, alternatives have been sought to simplify the measurement of insulin resistance. In recent years, several markers have been proposed for the screening, diagnosis, and therapeutic monitoring of insulin resistance. However, all have problems that limit their use to research studies. None succeeds in integrating the global assessment of the metabolic abnormalities that may increase risk for developing type 2 diabetes (T2DM) and metabolic syndrome (MS).

Compared to other classical insulin resistance indexes, quantitative insulin sensitivity check index (QUICKI) was reported to have the advantage of being applicable to wider ranges of insulin sensitivity and more reproducible [61]. It has been showed that QUICKI was among the most accurate and useful surrogate indexes for determining insulin sensitivity in humans [62]. However, QUICKI and insulin action do not correlate highly, particularly in individuals with mildly insulin resistance, impaired glucose tolerance or elderly patients with poorly controlled T2DM [63]. Moreover, QUICKI is less robust for early diagnosis of insulin resistance in persons without T2DM or MS [63]. QUICKI has low sensitivity for detecting insulin resistance in lean individuals with beta cell dysfunction [63]. QUICKI use fasting glucose levels in their calculations and fasting glucose levels are steady-state levels that are not a reflection of glucose utilization after a glucose load [62]. Also, QUICKI reflects hepatic insulin resistance only, not insulin resistance at peripheral tissues [63].

The recently developed HOMA-AD was a more accurate indicator for assessing insulin resistance than the HOMA-IR [64]. HOMA-AD is a modified version of homeostasis model assessment of insulin resistance (HOMA-IR) index which calculated from the product of serum insulin and plasma glucose levels divided by serum adiponectin levels [64]. Modification of HOMA-IR with adiponectin levels resulted in an index exhibiting a good correlation with M-values even in diabetic patients with moderate hyperglycemia [64]. In addition, Zaletel et al. showed that the adiponectin derived index correlated best with the euglycemic hyperinsulinemic clamp derived sensitivity index compared to other surrogate measures of insulin resistance including HOMA-IR, QUICKI, fasting glucose/insulin ratio or McAuley index [65]. A recent electron spin resonance study revealed adiponectin might has a close correlation with rheological behavior and microcirculation in hypertension [66]. Moreover, adiponectin may be a marker for global metabolic status including insulin resistance and metabolic syndrome [67].

It is well-established that insulin resistance in adipose tissue will lead to elevated serum resistin levels and reduced serum adiponectin levels [10]. Lipolysis plays a role in the developing of insulin resistance in healthy subjects, with an estimated overall contribution of approximately 39% [68]. Increased lipolysis in adipose tissues was associated with elevation of systemic free fatty acids and insulin resistance [69]. Adiponectin in physiological concentrations inhibits spontaneous as well as catecholamine-induced lipolysis [70]. Resistin induces lipolysis and re-esterification of triacylglycerol stores, and increases cholesteryl ester deposition, in human macrophages [71]. Therefore, inclusion of adiponectin and resistin into the QUICKI formula can be beneficial and can increase its detection power by including those subjects with peripheral insulin resistance, especially in view of the following: increased fasting resistin levels [71] and reduced fasting adiponectin levels [70] could reflect insulin resistance earlier than hyperglycemia since lipolysis was more sensitive to insulin than glucose utilization; a small increase in resistin [72] and decrease in adiponectin levels [73] in healthy individuals were reported to induce insulin resistance; insulin resistance of lipolysis induced by adiponectin and resistin were suggested as explaining a large variation in insulin sensitivity of glucose disposal in lean individuals [69].

It has been well-reported that adiponectin [67] and resistin [6] are promising biomarkers of insulin resistance. A recent clinical trial revealed pioglitazone plus vildagliptin treatment improved both adiponectin and resistin levels and might effective in preserving beta-cell function, and in reducing insulin resistance and inflammatory state parameters in subjects with poorly controlled T2DM [74]. Moreover, the imbalance in deleterious and protective adipokines including adiponectin and resistin plays pivotal roles in the development and progression of pancreatic beta-cell dysfunction under insulin-resistant conditions [75]. Adiponectin and resistin levels were also strongly correlated with the key metabolic endpoints of T2DM and MS as well as insulin sensitivity in the present study (Table 3 and Table 4). Therefore, we generated a modified version of QUICKI, the novel IR_AR _index by taking account into adiponectin and resistin levels. The IR_AR _index clearly had the narrowest and most favourable distribution of residuals among the surrogate indexes (Table 6 and Figure 4). The IR_AR _index may has higher precision, consistency, reproducibility and robustness than classical surrogate indexes (Table 6 and Figure 4). Thus, we hypothesized that the IR_AR _index may be a more appropriate model of insulin sensitivity than other surrogate indexes in Malaysian men.

The reported values for the definition of insulin resistance vary widely. A World Health Organization (WHO) consensus group concluded that the insulin sensitivity index of the lowest 25% of a general population can be considered as an insulin resistance state [76]. The European Group for the Study of Insulin Resistance take a more restricted view, defining insulin resistance as the insulin sensitivity index of the lowest 10% of a nonobese, nondiabetic, normotensive white population [77]. Therefore, further investigation or replicate studies are required to validate the suggestive reference range or minimum cut-off values of insulin sensitivity for the IR_AR _index in Malaysian men. In addition, long-term prospective studies are required to determine the actual reference range or minimum cut-off values of the novel IR_AR _index for insulin resistance assessment in the general population. The novel IR_AR _indexes give an opportunity to implement and use of this index in the daily clinical practice for screening persons with increased risk of future development of T2DM and MS due to insulin resistance. It is also very useful for monitoring the diseases progression and therapeutic response. Furthermore, it will allow early treatment or delay the onset of long-term severe complications such as cardiovascular risk.

### Strengths and limitations

This represent a first attempt to study the interaction effect of adiponectin and resistin in the modulation of the key metabolic endpoints of T2DM and MS. Our samples comprised of Malay, Chinese and Indian subjects from Malaysia, which represented a major segment of the Asian population. The covariates in term of ages and ethnicity were in homogeneity and were matched with the case-control groups (Table 1). Most potential confounders were carefully controlled for, which limits the possibility of residual confounding effect. Given the well-established difference in circulating adiponectin [8,67] and resistin [6] levels between men and women, our samples were only comprised male subjects to avoid the confounding effect of gender. Clinical measurements were taken under standardized protocol and biomarkers were measured using assays with good precision (Additional file 1, Figure S1 and Additional file 1, Supplementary Methods). Although it has been reported that various definitions of MS hold different predictive powers in detecting pathological levels of key adipocytokines such as hypoadiponectinemia and hyperresistinemia, the International Diabetes Federation (IDF) definition is quantitatively more powerful than its counterparts in terms of prevalence [4,78]. The IDF definition incorporates ethnicity by providing different criteria for the MS in different ethnic groups [4]. Moreover, the IDF definition is the most updated and globally accepted definition for MS [4].

Nevertheless, this study had limitations. The findings apply mainly to Malaysian men and may not be widely generalizable because of the homogeneity of the study population. Since this is not a prospective study, this study may have reverse causation due to possible effects of T2DM and MS on adiponectin and resistin levels. The results were based on single measurements of the adipokines and therefore may not reflect long-term exposure to these hormones. Although adjusted for known confounding factors, residual confounders imperfectly measured or unmeasured cannot be excluded.

We measured total adiponectin and not the high molecular weight fraction, which has been proposed to have substantially more potent effects on hepatic insulin sensitivity compared with total adiponectin [79]. However, a recent study showed that total and high-molecular-weight (HMW) adiponectin have similar utility for the identification of insulin resistance and metabolic disturbances [67]. This suggested that total adiponectin levels may provide clinical information of the same diagnostic value as HMW adiponectin [67]. It has been reported that the low molecular weight form of resistin displays significantly increased bioactivity [14]. However, the high molecular weight hexamer of resistin is predominant in the human circulation [14]. Moreover, the measurement of total adiponectin and resistin are better standardized, cheaper and more accessible than the high-molecular-weight adiponectin and the low-molecular-weight resistin. Thus, our findings may stimulate the use of adiponectin and resistin in clinical and epidemiological settings.

The euglycemic hyperinsulinemic clamp technique must be used as a gold standard index of insulin resistance to validate the robustness of the IR_AR _index before their widespread use. Further studies are required to investigate whether the superior predictive power of the AR and IR_AR _indexes demonstrated in the present study translates into a significant clinical benefit. The AR and IR_AR _indexes are unlikely to be cost-effective for short-time administration of these indexes. However, the AR and IR_AR _indexes are good indicator for long-term metabolic status. Thus, it will be very cost-effective for long-time administration since the frequency of monitoring the disease progression and therapeutic response periodically will be greatly reduced. A long-term prospective research is needed to reveal the predictive value of the AR and IR_AR _indexes for insulin resistance in association with T2DM and MS, and to find their optimal cutoff values for future risk assessment and disease prevention. Moreover, the normal AR and IR_AR _indexes range need to be established for each laboratory with an appropriate control group because of significant inter-laboratory variations in insulin and adipokines (including adiponectin and resistin) determinations and/or possible differences in various populations.

## Conclusions

The novel AR and IR_AR _indexes are cost-effective, precise, reproducible and reliable integrated diagnostic biomarkers of insulin sensitivity for screening subjects with increased risk of future development of T2DM and MS. These surrogate indexes are useful for early diagnosis of insulin resistance, T2DM and MS in the daily clinical practice and for large-scale clinical investigation.

## Competing interests

The authors declare that they have no competing interests.

## Authors' contributions

CHL carried out the laboratory works, collected the data and samples, performed the statistical analysis, and wrote the manuscript. SM participated in the design and coordination of the study and helped to edit the manuscript. All authors read and approved the final manuscript.

## Supplementary Material

Additional file 1 **Supplementary Information**. 1. Supplementary Methods: Subjects Determination of the anthropometric clinical and metabolic parameters 2. Supplementary Table Table S1. Mathematical equations for each insulin resistance (IR) index 3. Supplementary Figure and Figure Legend Figure S1. Standard curve for ELISA adiponectin and resistin Click here for file
