# Supplementary material for: Novel adiponectin-resistin (AR) and insulin resistance (IRAR) indexes are useful integrated diagnostic biomarkers for insulin resistance, type 2 diabetes and metabolic syndrome: a case control study
Source: Cardiovasc Diabetol. 2011 Jan 21;10:8. doi: 10.1186/1475-2840-10-8 (PMC3036610; doi:10.1186/1475-2840-10-8)
Supplement: Additional file 1 — Supplementary Information. 1. Supplementary Methods: Subjects Determination of the anthropometric clinical and metabolic parameters 2. Supplementary Table Table S1. Mathematical equations for each insulin resistance (IR) index 3. Supplementary Figure and Figure Legend Figure S1. Standard curve for ELISA adiponectin and resistin [file 1475-2840-10-8-S1.PDF]

SUPPLEMENTARY INFORMATION

1. Supplementary Methods

Subjects. Blood samples were collected between 8:00 A.M. and 11:00 A.M. after an overnight fast (~10-12 hours). The subjects were also free of vigorous exercise for 24 hours. They were asked not to take their diabetes, hypertensive and dyslipidemia medications or to perform insulin injection for 24 hours before the visit. Blood samples were drawn from an antecubital vein with a 22G x 1'' (0.7mm x 25mm) needle (Bd Vacutainer, Franklin Lakes, USA) without venous stasis into each Lavender closure with additive K2 EDTA blood collection tube (EDTA tube), SST blood collection tubes, red closure with additive clot activator blood collection tube (plain tube), and grey closure with additive potassium oxalate and sodium fluoride blood collection tube (glycolytic inhibitor tube) size 13mm x 75mm, respectively (Bd Vacutainer, Franklin Lakes, USA). The whole blood containing plain tubes and SST tubes were centrifuged at 2700 rpm for 10 minutes to separate sera from blood cells. The sera from plain tubes were used for the insulin, adiponectin and resistin levels measurement while the sera from SST tubes were used for the lipid profile (total cholesterol, HDL cholesterol, LDL cholesterol, and triglyceride levels) determination. The plasma from glycolytic inhibitor tubes were used for glucose levels measurement. Meanwhile, the whole blood containing EDTA tubes were used for HbA1C levels measurement. Sera were stored at -20°C for subsequent biochemical assays.

Determination of anthropometric clinical and metabolic parameters. Fasting serum total adiponectin and resistin levels were determined with AssayMax Human Adiponectin ELISA Kit (AssayPro, St. Charles, Missouri, USA) and AssayMax Human Resistin ELISA Kit (AssayPro, St. Charles, Missouri, USA), respectively. For the adiponectin assayed, the intra-assay and inter-assay coefficients of variation were 4.1% and 7.2%, respectively with a

sensitivity of 0.5ng/ml (Additional file 1, Figure S1A). Meanwhile, the intra-assay and inter-assay coefficients of variation were 4.0% and 7.2%, respectively with a sensitivity of <100pg/mL for the resistin assayed (Additional file 1, Figure S1B).

Fasting serum insulin, total cholesterol, LDL cholesterol, HDL cholesterol, triglyceride, plasma glucose, and whole blood HbA1C levels were determined from the Clinical Diagnostic Laboratory of the University Malaya Medical Centre (UMMC). Fasting serum insulin levels were measured by an ADVIA Centaur assay (Siemens Medical Solutions Diagnostics, Tokyo, Japan) with a sensitivity of 0.5mU/L, and intra-assay and inter-assay coefficients of variation (CV) of 4.6 and 5.9%, respectively. Fasting serum total cholesterol, triglyceride and plasma glucose levels were measured using an automated analyzer (Siemens Healthcare Diagnostics Inc., Newark, USA). The intra-assay and inter-assay coefficients of variation were 0.84% and 1.3%, respectively with a sensitivity of 1.3mmol/L (50mg/dL) for the total cholesterol assayed. Meanwhile, the intra-assay and inter-assay coefficients of variation were 0.4% and 1.3%, respectively with a sensitivity of 0.17mmol/L (15mg/dL) for the triglycerides assayed. The intra-assay and inter-assay coefficients of variation were 0.6% and 1.6%, respectively with a sensitivity of 0mmol/dL (1mg/dL) for the glucose assayed.

Fasting serum HDL cholesterol levels were measured using an automated analyzer (Dade Behring Inc., Newark, USA). The intra-assay and inter-assay coefficients of variation were 1.57% and 2.00%, respectively with a sensitivity of 0.26mmol/L (10mg/dL) for the HDL cholesterol assayed. LDL cholesterol levels were calculated using the Friedewald formula as following: LDL cholesterol (mmol/L) = total cholesterol (mmol/L) – HDL cholesterol (mmol/L) – [triglycerides(mmol/L) / 2.2]. LDL cholesterol levels were not calculated if the sample triglyceride levels were ≥4.5 mmol/L because the Friedewald equation for the LDL cholesterol

estimation was not calibrated. Lastly, the whole blood Hemoglobin A1c (HbA1c) levels were determined by using COBAS INTEGRA Hemoglobin A1C (HbA1C) System (Roche Diagnostics Corporation, Indianapolis, Indiana, USA). The intra-assay and inter-assay coefficients of variation were 4.7% and 10.3%, respectively with a sensitivity of 3% for the HbA1C assayed.

Blood pressure was measured by Omron M5 Automatic Blood Pressure Monitor (OMRON Corporation, Kyoto, Japan) with an accuracy of ±3mmHg. The body mass index (BMI) was calculated using the following equation: BMI= weight(kg)/[height(m) x height(m)]. Meanwhile, the waist-to-hip ratio (WHR) was calculated using the following equation: WHR = waist(cm)/hip(cm).

2. Supplementary Table

Table S1 Mathematical equations for each insulin resistance index

Insulin resistance indexes	Mathematical equations	Symbolizes (Units)
HOMA-IR	$\frac{I_0 \times G_0}{22.5}$	I_0 = fasting serum insulin levels (µU/mL)<br>G_0 = fasting plasma glucose levels (mmol/L)
QUICKI	$\frac{1}{\log(I_0) + \log(G_0)}$	I_0 = fasting serum insulin levels (µU/mL)<br>G_0 = fasting plasma glucose levels (mg/dL)
Bennett	$\frac{1}{\ln(I_0)\ln(G_0)}$	I_0 = fasting serum insulin levels (µU/mL)<br>G_0 = fasting plasma glucose levels (mg/dL)
McAuley (1)	$\exp^{2.63-0.28\ln(I_0)-0.31\ln(TG_0)}$	I_0 = fasting serum insulin levels (µU/mL)<br>TG_0 = fasting serum triglyceride levels (mmol/L)
McAuley (2)	$\exp^{3.29-0.25\ln(I_0)-0.22\ln(BMI)-0.28\ln(TG_0)}$	I_0 = fasting serum insulin levels (µU/mL)<br>BMI = body mass index (kg/m^2)<br>TG_0 = fasting serum triglyceride levels (mmol/L)

Note: HOMA-IR = homeostasis model assessment of insulin resistance; QUICKI = quantitative insulin sensitivity check index.

3. Supplementary Figure and Figure Legend

A)

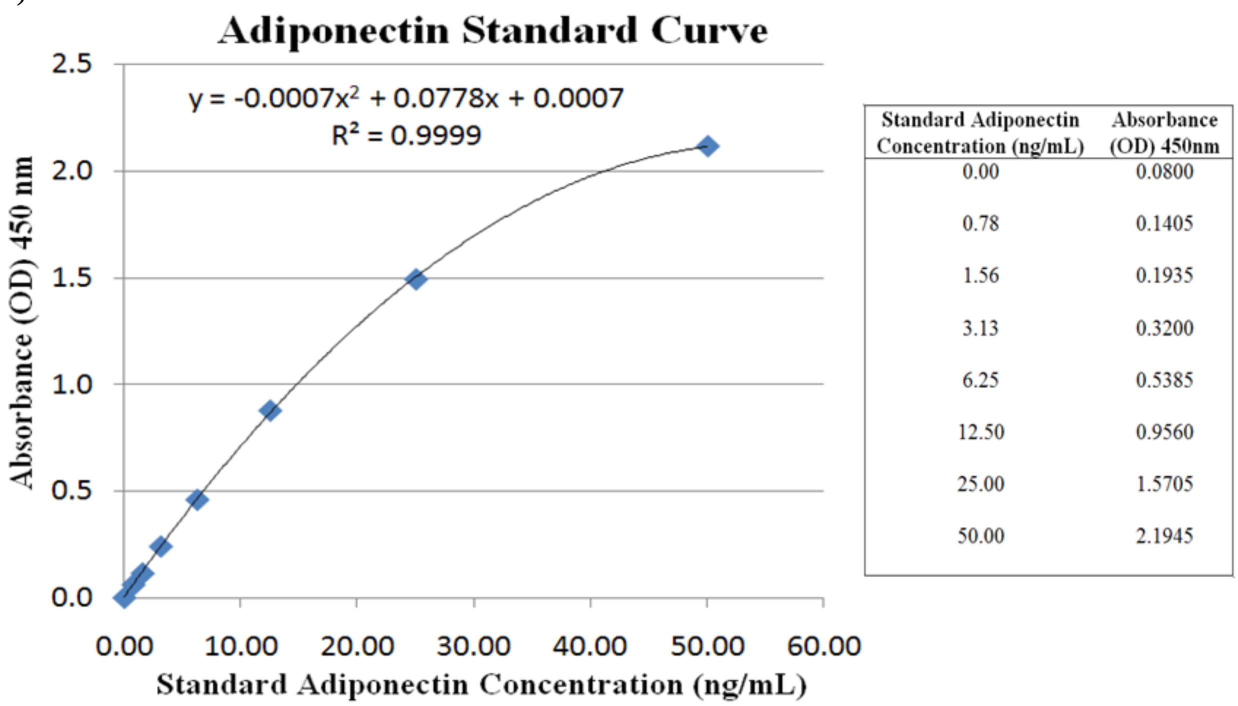

Standard Adiponectin Concentration (ng/mL)	Absorbance (OD) 450nm
0.00	0.0800
0.78	0.1405
1.56	0.1935
3.13	0.3200
6.25	0.5385
12.50	0.9560
25.00	1.5705
50.00	2.1945

B)

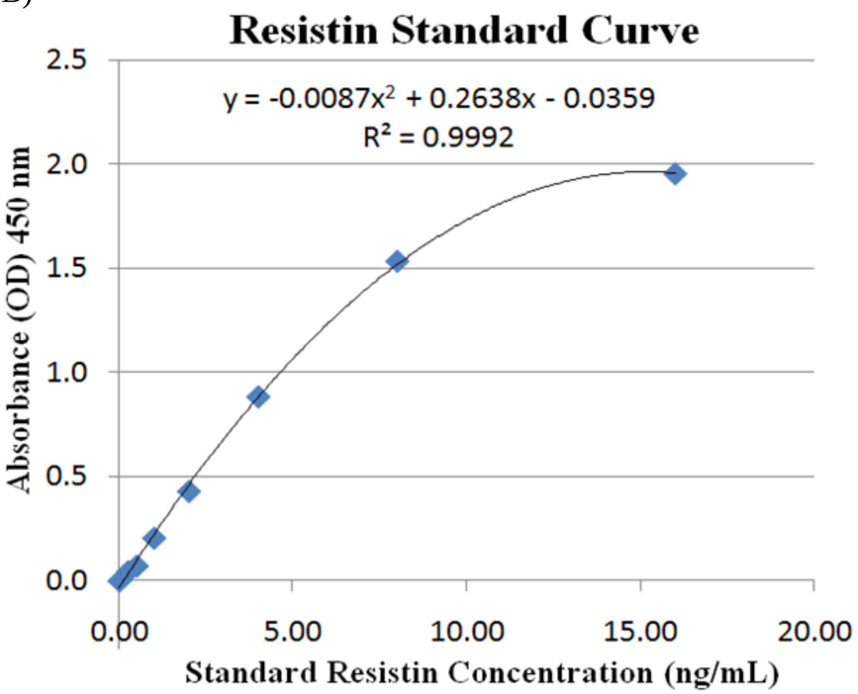

Standard Resistin Concentration (ng/mL)	Absorbance (OD) 450nm
0.00	0.0735
0.25	0.1175
0.50	0.1445
1.00	0.2785
2.00	0.5025
4.00	0.9560
8.00	1.6065
16.00	2.0260

Figure S1. Standard curve for ELISA adiponectin and resistin. (A) Adiponectin standard curve. (B) Resistin standard curve.
